# Effect of Turmeric–Boswellia–Sesame Formulation in Menstrual Cramp Pain Associated with Primary Dysmenorrhea—A Double-Blind, Randomized, Placebo-Controlled Study

**DOI:** 10.3390/jcm12123968

**Published:** 2023-06-11

**Authors:** Divya Agarwal, Priyanka Chaudhary

**Affiliations:** Smt. Meva Chaudhary Hospital, Opp. MLB Medical College, Jhansi 284128, India

**Keywords:** dysmenorrhea, menstrual cramp, pain, turmeric, boswellia, sesame

## Abstract

Primary dysmenorrhea is a common menstrual disorder that significantly impacts women’s quality of life, productivity, and healthcare utilization. In this randomized, double-blinded, placebo-controlled trial, sixty women with primary dysmenorrhea were randomly divided into two groups with thirty participants each, and were allocated either turmeric–boswellia–sesame formulation (treatment) or placebo. The participants were advised to take two softgels of 500 mg as a single dose of allocated study intervention (total dose 1000 mg) when their menstrual pain reached 5 or more on a numerical rating scale (NRS). Menstrual cramp pain intensity and relief were evaluated every 30 min post-dose until 6 h. Results indicated a promising role of turmeric–boswellia–sesame formulation for menstrual pain relief compared to the placebo. The mean total pain relief (TOTPAR) of the treatment group (18.9 ± 0.56) was found to be 12.6 times better than the placebo group (1.5 ± 0.39). The NRS analysis showed that there was a statistically significant difference in pain intensity between the treatment and placebo groups (*p* < 0.001) at every timepoint. Additionally, the sum of pain intensity difference at 6 h (SPID6) of the treatment group (34.32 ± 1.41) showed a significant difference (*p* < 0.0001) and was 20.19 times better when compared to placebo (1.7 ± 0.56). Based on the study results, the turmeric–boswellia–sesame formulation exhibited remarkable menstrual pain relief as compared to the placebo.

## 1. Introduction

Dysmenorrhea, often known as painful menstruation, is characterized by intense, excruciating cramping in the lower abdomen that is frequently accompanied by additional symptoms such as sweating, headache, nausea, vomiting, diarrhea, and trembling that appear right before or during menstruation [1,2]. Primary dysmenorrhea is painful menstruation with no evidence of hormonal or anatomic (pelvic) pathology. It is one of the most prevalent and important complaints of women in their reproductive age [3]. The burden of dysmenorrhea is larger than that of any other gynecological complaint [4], and it affects more than half of all women of reproductive age regardless of age, nationality, or socioeconomic status [5,6]. In primary dysmenorrhea, the pain usually begins a few hours before or right after menstruation and lasts about 48–72 h [7]. Women with primary dysmenorrhea experience lower physical activity, diminished work productivity, and a reduced quality of life [8].

Primary dysmenorrhea’s underlying etiology is not fully understood. However, it has been determined that an excess of uterine prostaglandins, particularly PGF2a and PGF2, is responsible for an increase in uterine tone and high-amplitude contractions [9]. Prostaglandin levels are higher in women with dysmenorrhea, and are at their maximum during the first two days of menstruation [10]. Progesterone regulates prostaglandin synthesis; prostaglandin levels rise right before menstruation, when progesterone levels fall [9,11]. The perception and degree of pain, however, may be influenced by a variety of other factors in addition to endocrine ones [12]. Menstrual pain must be treated to reduce long-term effects and it may make women more susceptible to other chronic pain issues later in life [9].

Due to its significance, various treatments have been employed to lessen the effects of dysmenorrhea, including pharmacological and nonpharmacological treatment approaches such as nonsteroidal anti-inflammatory medicines (NSAIDs), herbal, nutritional, yoga, meditation, and acupuncture [13]. Amongst all, non-steroidal anti-inflammatory medicines (NSAIDs) are a mainstay, either alone or in combination with oral contraceptives or progestins [14]. NSAIDs prevent prostaglandins’ production by inhibiting the enzyme cyclo-oxygenase (COX) [15,16,17,18]. It has been shown that decreased prostaglandin production lessens the force of uterine contractions, easing women’s discomfort [15]. However, NSAIDs may negatively impact the kidneys, liver, and circulatory system, raising the risk of thromboembolic problems [16,18]. A total of 20% to 25% of women do not respond to or are inappropriate for conventional treatment for primary dysmenorrhea [19]. Herbal remedies could be an effective solution for primary dysmenorrhea. Although numerous studies have been conducted on the analgesic effect of plant extracts, there is a rising demand to find an effective remedy for primary dysmenorrhea.

Ancient medicine utilized the powdered rhizome of *Curcuma longa* L. (*Zingiberaceae*), also known as turmeric, to treat inflammation and wound healing [20]. Curcumin was reported to be effective in mood and behavioral changes related to premenstrual syndrome [21]. Boswellia serrata has demonstrated significant pharmacological efficacy in treating chronic inflammatory disorders such as rheumatoid arthritis, chronic bronchitis, asthma, ulcerative colitis, and Crohn’s disease [22,23]. Sesame has a known history of use in the ancient system of medicine related to menstrual irregularities [24]. Turmeric–boswellia–sesame formulation is a proprietary blend of turmeric and boswellia extract with sesame oil, and this specific formulation serves as an alternative treatment for menstrual cramp pain. The present study was designed to investigate the effectiveness of turmeric–boswellia-sesame formulation for primary dysmenorrhea.

## 2. Materials and Methods

### 2.1. Study Design

This was a randomized, double-blinded, placebo-controlled study in adolescent and adult female participants with primary dysmenorrhea of at least moderate pain. The study was conducted in accordance with the Declaration of Helsinki at Smt. Meva Chaudhary Hospital, Jhansi after obtaining approval from Nirmal Hospital Institutional Ethics Committee, Jhansi, Uttar Pradesh (date of approval: 19 May 2022). No amendments on the approved study protocol (protocol code AN-05PFK0422H4-WES09) were performed after starting the study. The study was prospectively registered in Clinical Trial Registry, India (CTRI/2022/05/042916).

### 2.2. Participant Selection

Female participants who provided a voluntary informed consent and met study inclusion and exclusion criteria were enrolled. Healthy female participants between 18 and 35 years of age having regular menstrual cycles that typically occur between every 21 to 35 days, with a self-reported history of primary dysmenorrhea and at least moderate menstrual cramp pain (based on the categorical pain intensity scale 0–3; 0 = none, 1 = mild, 2 = moderate, 3 = severe), and who provided voluntary informed consent showing the willingness to participate in the study were included in the study.

The following comprises the exclusion criteria: participants having a known allergy to any of the ingredients in any of the study medication products; or with significant co-existing illnesses, including gastrointestinal, hepatic, renal, neurologic, cardiovascular, psychiatric, endocrine, respiratory, and surgical procedure, or other condition that, in the investigator’s judgment, contraindicates administration of the study medication; or with a current or past history of one or more of the following conditions: secondary dysmenorrhea, pelvic inflammatory disease, urinary tract infection (currently acute or recurrent (defined as more than three per year)), adnexal masses, uterine fibroids, endometriosis, and adenomyosis that in the opinion of the investigator would impact participant safety and/or the study data; or with an ongoing sexually transmitted disease (except for a history of genital herpes or human papillomavirus); or has abnormal vaginal discharge; participant requiring prescription analgesics, narcotic, non-NSAID (i.e., defined as oral use of 5 or more times per week for greater than 3 weeks); or has routinely taken OTC medications in excess of label-recommended instructions for the control of dysmenorrhea symptoms; who were taking mood-altering agents (e.g., antidepressants, sedatives, phenothiazines, or anti-anxiety agents); who did not agree to abstain from taking any analgesic and/or anti-inflammatory medication approximately 72 h prior to the anticipated treatment period and throughout the dosing/assessment period; who were pregnant, lactating, or less than 6 months postpartum; or using an oral contraceptive for less than 3 months, have been on an unstable dose within the last 3 months, or have switched from one oral contraceptive to another within the last 3 months of the study; or with a history of daily alcohol intake or drug abuse or a medical disorder, condition or history such that could impair the participant’s ability to participate or complete this study in the opinion of the investigator. All pain and anti-inflammatory medications, including supplements, topical heat or cold, and other products of topical application were discontinued approximately three days before the expected first day of menstruation and throughout the dosing/assessment period.

### 2.3. Study Intervention

Qualified participants were randomized into two groups in a 1:1 ratio to a single dose of two softgels of 500 mg test product (turmeric–boswellia–sesame formulation, Arjuna Natural Pvt. Ltd., Aluva, India) or placebo (two softgels of 500 mg) for one day. The active ingredients of the test product were turmeric extract (95%), 28% (containing NLT 26.6% total curcuminoids), Boswellia serrata extract, 10% (containing AKBA NLT 1%), and sesame oil, 62%. Investigational products (IP) were packed in small opaque bottles. The treatment and the placebo arms were concealed using an alphabet code. The allocation concealment randomization schedule was given to the pharmacist for serial dispensation. The investigator and the participants were blinded from the identity of the IP dispensed. The blinding was achieved using a placebo comparator with similar packaging and labelling.

### 2.4. Randomization Procedures

The randomization method used in this study was a balanced stratified randomization with 1:1 allocation and the sequences were generated using the software WinPepi, version 11.65. The master randomization list was prepared by an independent statistician and given to the pharmacist to dispense IPs. The randomization schedule and the IPs were under the restricted access of the pharmacist to prevent selection bias. The pharmacist dispensed the IPs serially according to the schedule and no other study staff were involved in the IP dispensation.

### 2.5. Pain Assessment

#### 2.5.1. Categorical Pain Intensity (0–3) (at Screening)

A categorical pain intensity scale (0–3) was used during screening for selecting the participants.

Category 0, 1, 2, and 3 represent ‘none’, ‘mild’, ‘moderate’, and ‘severe’, respectively. Participants having past menstrual pain ≥2 on the categorical pain intensity scale were considered for the study.

#### 2.5.2. Numerical Rating Scale for Pain (NRS) (0–10)

The NRS is an 11-point scale in which 0 represents ‘no pain’ and 10 represents the worst pain possible. The participants were asked to rate their pain intensity as a number from 0 to 10. The participant took the assigned study medication when menstrual cramp pain was ≥5 in severity using the 0–10 numerical rating scale (NRS) questionnaire and noted the time.

After dosing, the participant provided the pain intensity rating every 30 min up to 6 h post-dose for the assessment of the sum of pain intensity difference (SPID).

#### 2.5.3. Categorical Pain Relief Scale (0–4) (PRS)

The pain relief scale is a categorical scale having a positive progression from ‘No relief’ to ‘Complete relief’ where 0, 1, 2, 3, and 4, respectively, represent ‘no relief’, ‘a little relief’, ‘some relief’, ‘a lot of relief’, and ‘complete relief’. After dosing, the participant rated the pain relief every 30 min up to 6 h for the assessment of total pain relief (TOTPAR) at 6 h.

#### 2.5.4. Global Evaluation Assessment

The participant rated the effectiveness of the study medication in relieving menstrual cramp pain at 6 h post-dose or immediately at the first intake of rescue medication in a categorical scale from 0–4, where 0, 1, 2, 3, and 4, respectively, represent ‘poor’, ‘fair’, ‘good’, ‘very good’, and ‘excellent’.

### 2.6. Study Outcome Measures

The primary outcome measure of the study was the total pain relief scores (TOTPAR) at 6 h, using the categorical pain relief scale. TOTPAR is calculated as the sum of pain relief scores over a period of time. The other study outcome measures were summed pain intensity difference (SPID) over the 6 h study period (SPID 0–6) using 0–10 NRS. SPID was calculated as the sum of the differences between the current pain scores and baseline pain score over the study period.

Percentage max TOTPAR and percentage max SPID were calculated for each individual and categorized as <30%, 30–49%, 49–69%, and >70%. Maximum TOTPAR for an individual is the maximum relief score obtained multiplied by time in hours. Maximum possible SPID for an individual is the initial pain rating multiplied by the number of hours over which ratings were recorded. Values for TOTPAR and SPID of each individual were, respectively, converted into the percentage of maximum TOTPAR and the percentage of maximum SPID by dividing with the calculated maximum value of TOTPAR and SPID [25]. The effectiveness of study medication was assessed using a global evaluation categorical scale.

### 2.7. Evaluation of Safety

Adverse event will be assessed as treatment emergent adverse events (TEAEs) throughout the study. AEs will be collected from screening throughout the treatment phase. Only treatment emergent AEs will be included, i.e., AEs that begin or worsen after the first dose of the study medication in the treatment phase. The number and percent of participants who experience any event will be displayed by treatment group.

### 2.8. Study Procedure

The study consisted of a twenty-one-day screening phase and a treatment phase (menstruation period). Qualified participants were randomized into one of two sequences—treatment or placebo.

#### 2.8.1. Screening Phase Visit 1 (Day-21 to -1):

Eligible female participants were selected within a screening period of 21 days prior to the start of the first menstruation (treatment phase). Participants were questioned in order to determine the severity of menstrual pain (at least moderate pain on a categorical pain intensity scale (0–3, 0 = none, 1 = mild, 2 = moderate, 3 = severe) in order to be included) experienced in past menstrual cycles prior to randomization in the study. Participants having pain ≥ 2 on categorical pain intensity scale were considered for the study.

After the successful completion of screening procedures, participants were randomized into one of two groups as per the allocation concealment randomization list accessed only by the pharmacist. In addition, each participant received either test product or placebo, study documents and pregnancy tests were performed before the intake of study medication. The participants received training on the use of the study documents in addition to instructions on how to take their assigned medication.

#### 2.8.2. Treatment Phase

After completion of screening and randomization, participants started the treatment phase when their menstruation begins. This part of the treatment phase varies from participant to participant depending on the duration of their menstrual cycle (in days) and when the menstrual cycle started with respect to randomization.

Participants were instructed to use their study documents when they think they are close to approaching the first day of their menstrual cycle. Participants would discontinue the use of all pain medications, including supplements, topical heat or cold, and other products of topical application, approximately three days before the expected first day of menstruation and throughout the treatment/assessment period. The participants took the assigned study medication when menstrual cramp pain was ≥5 in severity using the 0–10 numerical rating scale (NRS) questionnaire. Before dosing, the participants completed a baseline (immediately before dosing) 0–10 NRS menstrual cramp pain intensity evaluation.

After taking the study medication, the participants completed the pain/pain relief assessment (0–10 NRS and the 0–4 categorical pain relief scale) at 30 min time intervals using the study documents over the next 6 h time period. At 6 h post-dose, the participants completed the global evaluation of their overall satisfaction with the effectiveness of the study medication. If the participant decides to take rescue medication prior to the 6 h, standard of care was offered by the principal investigator and the participant were instructed to complete the global evaluation prior to taking the rescue medication.

### 2.9. Statistical Analysis

In this pilot study, a specific sample size calculation was not performed and a fixed sample size of 60 participants was selected. Shapiro–Wilk test of normality was conducted for all variables in this study to understand the distribution of data. Based on the data distribution, appropriate parametric and non-parametric tests were conducted. NRS-derived endpoint SPID was analyzed using the Mann–Whitney U test to compare between placebo and treatment. Variability between the treatment and placebo was estimated from the least square mean (LSM) with 95% confidence intervals (CIs) and associated two-sided Bonferroni adjusted *p*-values. The pain intensity difference (PID) at different timepoints from baseline was analyzed using a mixed model repeated measures with fixed effects for treatment, time, and treatment–time interaction. TOTPAR between the treatment and placebo groups were analyzed using the Mann–Whitney U test. All statistical analyses were performed using NCSS v.2021 and R version 4.1.0 (R team, Vienna, Austria, 2021). Global evaluation data is a form of assessment that integrates the investigator’s or patient’s overall impression about the change in the state of the treatment, and is usually a scale of ordered categorical ratings. Hence, this was analyzed by ANOVA after assigning numerical scores to the ordered categories [26].

## 3. Results

In the study, 64 female participants were screened and 60 eligible participants were enrolled in the study. They were randomized into two groups with 30 participants each. There were no protocol deviations or violations in the study. This was a single-day single-dose study and there was a 100% treatment compliance for all study participants. The participants were directly observed during the study period and assessments were taken half-hourly post-dose. All the participants in the study completed the half-hourly pain survey and the rate of questionnaire compliance at each timepoint was 100%. There were no adverse events reported in the study and no dropouts in this study. None of the participants in the study received rescue medications during the study period. Demographics and baseline characteristics of the study groups are mentioned in Table 1.

The study results showed that there was significance relief in menstrual pain with treatment when compared to placebo. The mean TOTPAR at 6 h was 18.9 ± 0.56 for the treatment group and 1.5 ± 0.39 for the placebo. The PRS analysis showed that there was a statistically significant difference in pain relief between the treatment group and placebo (*p* < 0.001). Mean TOTPAR of the treatment group was 12.6 times better than placebo (Table 2).

**Figure 1:**
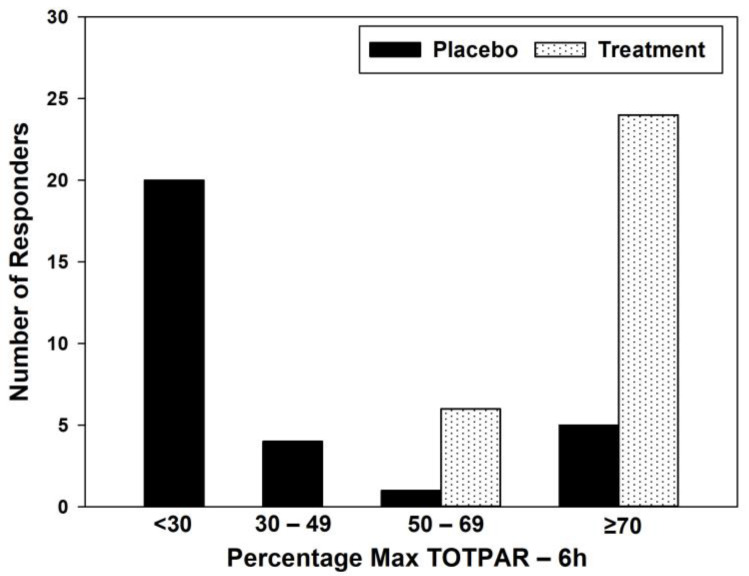
Responder profile for % max TOTPAR.

A total of 80% of participants in the treatment group had a % max TOTPAR equal to or more than 70%, whereas in the placebo group, 66.67% of the participants had a % max TOTPAR of less than 30% (Table 3) (Figure 1).

The number needed to treat to achieve a total pain relief of more than 50% of maximum achievable TOTPAR is 1.25 since all participants (100%) achieved a TOTPAR of more than 50% of max TOTPAR, whereas only 20% of the participants achieved this in the placebo group.

Menstrual pain intensity significantly reduced with treatment. SPID at 6 h of treatment group showed a significant difference (*p* < 0.0001) when compared to placebo and 20.19 times better than placebo (Table 4).

**Figure 2:**
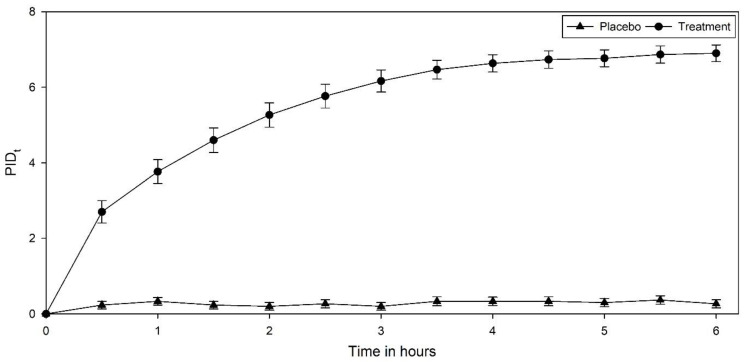
Pain intensity difference from baseline at different timepoints over a period of 6 h with treatment and placebo.

The LSM difference of treatment from placebo showed a statistically significant difference until the end of the study at the specified timepoints. The NRS analysis showed that the treatment reduced menstrual pain intensity, which was statistically significant. Least-square-mean pain intensity difference (LSM PID) of treatment and placebo are detailed in Table 5 and represented in Figure 2.

**Figure 3:**
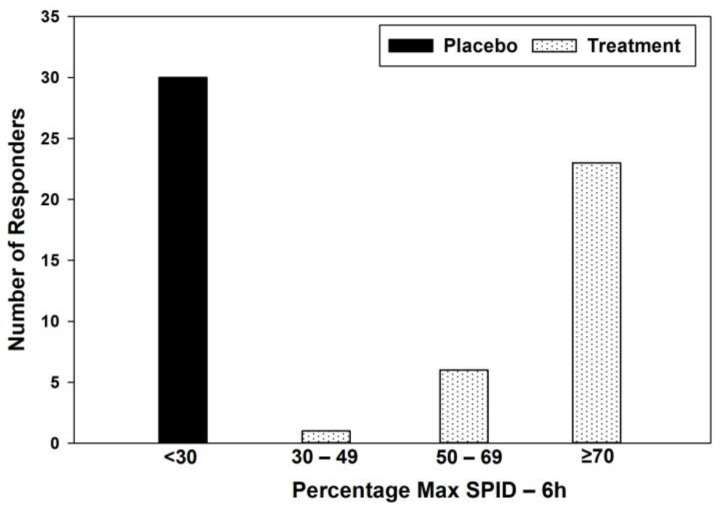
Responder profile for % max SPID.

A total of 76.67% of participants in the treatment group had ≥70% max SPID, whereas in the placebo group, 100% of the participants had <30% of max SPID (Table 6) (Figure 3).

**Figure 4:**
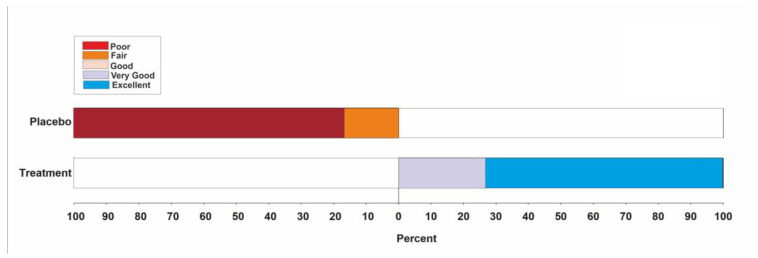
Global evaluation assessment.

Based on the global evaluation categorical scale, 73.3% in the treatment group reported that the formulation is an excellent pain reliever, whereas in the placebo group 83.3% reported poor pain relief (Table 7) (Figure 4).

## 4. Discussion

Menstruation is a complex cycle controlled by female hormones. The four phases of the menstrual cycle are menstruation, follicular, ovulatory, and luteal phases. If the mature egg that is released during ovulation is not fertilized, estrogen and progesterone levels eventually drop to their lowest points in the late luteal phase, which signals the body to shed the lining of the uterus. It also signals to the body that it is time to stimulate the release of prostaglandins, the hormone-like compounds that help the blood flow down the uterus.

Primary dysmenorrhea is caused by excessive uterine contraction caused by high numbers of prostaglandins, the hormone-like compounds that cause the uterus to contract in order to shed its lining. The smooth muscles in numerous nearby tissues contract as a result of this prostaglandin. Colicky pains, spasmodic and labor-like symptoms in the lower abdomen, and lower back discomfort are all prompted by uterine smooth muscle contractions and are indicative of dysmenorrhea. Additionally, the gastric and intestinal smooth muscles contract as a result of prostaglandin release, which can produce nausea, vomiting, and diarrhea [27,28,29]. Prostaglandins also trigger the shedding of the lining of the uterus, endometrium, by restricting the oxygen supply in the cells of this lining. These cells die and this is what is shed during menstruation.

In animal studies, it was found that high levels of estrogen caused the uterus to contract more strongly by producing more prostaglandins [30,31], which may contribute to stronger levels of menstrual pain. The secretion of prostaglandins into the uterine tissue, especially in the anti-inflammatory mediators such as PGF2 and PGE2 plays a major role in uterine contractions [32]. The uterus prostaglandin production is enhanced by estrogen, immediately after the menstruation’s start reaches its peak. Progesterone inhibits prostaglandin synthesis until the very start of menstruation. In the endometrium, PGE2 and PGF2α predominate. PGE2 act as a potent platelet disperser and vasodilator, while PGF2α is a mediator and trigger for pain sensation and a strong stimulator of smooth muscle contraction. PGF2α also has an important pro-inflammatory function, while PGE2 is a key mediator of inflammation and pain and especially in chronic inflammatory [25]. Both PGE2 and PGF2α concentrations are higher in the menstrual fluid of women with dysmenorrhea, especially during the first day of menstrual flow in comparison with women with a painless period. According to these findings, prostaglandin suppression is considered as the most effective relief of primary dysmenorrhea [9]. The inhibition of prostaglandin synthesis is the main mechanism of NSAIDs. Mefenamic acid, highly effective nonselective COX inhibitor from NSAIDs family, inhibits the binding of PGE2 to its receptor rather than reducing prostaglandin synthesis; however, it indicates higher levels of gastrointestinal side effects [25]. Management of dysmenorrhea can be performed pharmacologically and non-pharmacologically. Pharmacological treatment options include taking analgesic medications, hormone therapy, prostaglandin nonsteroidal drugs, and lactical canal lactation; nonpharmacological options for treating menstrual pain include vitamin E supplements, acupuncture, hypnotherapy, and herbal products that have been believed to be helpful [33]. Today, a variety of methods are used to treat and manage pain and symptoms, including localized heat, medication, thiamin, vitamin E, fish oil supplements, acupuncture, and transcutaneous nerve stimulation. Similar to many chemical treatments, these pharmaceuticals, such as ibuprofen and mefenamic acid, have side effects. The side effects are noticeable, especially when synthetic medications are prescribed for an extended period of time. The adverse effects of prostaglandin synthesis inhibitors include nausea, stomach discomfort, ulcers, gastrointestinal problems, renal papillary necrosis, and renal blood flow. The use of complementary therapies, such as herbs or nutrients in the treatment of primary dysmenorrhea or associated problems, has attracted particular attention due to the side effects of these medications [34].

Okuvan et al. found that the addition of turmeric powder (1 g/day) to standard treatment naproxen (750 mg/day) during menstrual bleeding improved dysmenorrhea treatment outcomes. The decrease in VAS scores was significant in both groups; however, the percentage of VAS score decrease (61.7% vs. 76.8%) was significantly higher with the addition of turmeric powder to the NSAID naproxen [35]. In a clinical trial, 24 female students with dysmenorrhea consumed 15 g of sesame seeds days before and 3 days after the menstruation. Severity of dysmenorrhea was measured by a visual analog scale (VAS) in one period without consuming sesame and two periods with sesame consumption. Results showed that the pain of dysmenorrhea and the amount of consuming sedatives statistically decreased in periods with the consumption of sesame [36]. A pilot study conducted on 21 cases of oligomenorrhea found that sesame is effective in inducing menstrual bleeding in women with oligomenorrhea. Sesame powder at a dose of 60 g boiled in water was filtered and drunk, but participants reported the unpleasant taste of sesame and feelings of nausea. The study reported that two persons had decreasing dysmenorrhea [37]. The analgesic activity of water extract of myrrh (3.9 g/kg), frankincense (6.8 g/kg), and myrrh combined with frankincense (5.2 g/kg) were examined against oxytocin-induced dysmenorrhea in mice. The results showed that frankincense extract had no obvious effects on the writhing times of mice, but the combination of myrrh and frankincense reduced the writhing times and prolonged the latency period (*p* < 0.01). The results of an analysis of variance (ANOVA) stated that the effects of three extracts were remarkably different for reducing the writhing times, but showed no obvious difference on analgesic latency period [38].

The objective of the study was to evaluate the effectiveness of turmeric–boswellia–sesame formulation for the treatment of primary dysmenorrhea in comparison to placebo. With a single dose of 1000 mg of a turmeric–boswellia-sesame formulation, the mean TOTPAR assessed by PRS showed significant menstrual pain relief as compared to the placebo. In the treatment group, SPID at 6 h assessed by NRS showed a significant difference (*p* < 0.0001) when compared to placebo, indicating that the turmeric–boswellia–sesame formulation exhibited a remarkable reduction in menstrual pain as compared to the placebo. The global assessment shows that the overall efficacy and/or the effectiveness of a treatment was higher for the treatment group compared to the placebo.

Prostaglandin E2, also known as dinoprostone, is the most common and most biologically potent of mammalian prostaglandins. It is produced from PGH2 by prostaglandin E synthase, which has at least three forms that are structurally and biologically distinct. It appears that microsomal prostaglandin E synthase-1 (mPGES1) is the key enzyme in the formation of PGE2. Prostaglandin F2α may be formed directly from PGH2 by endoperoxide F reductase, but most often it is made from PGE2 by PGE 9-ketoreductase. Besides curcumin and sesame oil, the test product also contains Boswellic acids from Boswellia serrata. Suppression of PGE2 formation by boswellic acids via interference with microsomal prostaglandin E2 synthase-1 mPGES1 contribute to the anti-inflammatory effectiveness of boswellia and may constitute a biochemical basis for their use in alleviating pain in dysmenorrhea.

Sampson’s theory of retrograde menstruation is the leading theory and suggests that endometriosis is related to the backward flow of endometrial tissue via the fallopian tubes into the peritoneal cavity during menses. Retrograde menstruation occurs in 90% of menstruating women. It has been reported that curcumin was able to suppress the proliferation of endometrial cells by reducing estradiol level [39]. Bachmeier et al. demonstrated estrogenic effects of putative phytoestrogens at physiological concentrations and showed estrogenic effects of curcumin [40]. Estradiol is an important promoter of the growth of both eutopic and ectopic endometrium. The primary source of estradiol is the ovary, and estradiol has been recently found to be an effective regulator of endometriosis [41]. The effect of turmeric extracts on uterine contractions was investigated on strips isolated from mice uteri under the influence of estrogen. The results of the study indicated that these two herbal extracts may inhibit uterine contractions, which can be used as agents for the prevention of preterm labor. Their inhibition effects on contractions are caused by PGF2α, which could be useful for the treatment of dysmenorrhea [42]. Khayat et al., 2015, reported that curcumin administered for one week before and 3 days after the onset of menstrual bleeding for three consecutive menstrual cycles could improve the mood and behavioral symptoms of premenstrual syndrome by modulating neurotransmitters and attenuate the physical symptoms of menstruation cycle by inhibiting COX-2 enzyme (prostaglandin E2 synthesis) [43]. Unlike the NSAID, the test product, which is a unique composition of turmeric and boswellia extract with sesame oil via different pathways, alleviates dysmenorrhea and reduces the chance of presenting with endometriosis. Sesame (*Sesamum indicum* L.) oil in the test product contains the active compound sesamin. Sesamin, a sesame lignan, was recently reported to be converted by intestinal microflora to enterolactone, a compound with estrogenic activity. Enterolactone was the major metabolite of sesamin both in vivo and in vitro [44]. Sesame seeds are a rich source of lignans. In addition to sesamin, other lignans such as sesamolin, sesaminol, sesamolinol, and pinoresinol have been isolated from sesame seed or sesame seed oil [45]. Flaxseed lignans, isoflavones, and coumestans, have generally been categorized as the three major groups of phytoestrogens [46]. Enterolactone and enterodiol converted from flaxseed lignans by intestinal microflora are considered the agents responsible for estrogenic activity [47]. It was reported recently that sesame lignans are also metabolized efficiently to enterolactone [44]. Although it does not reduce serum estrogen, sesame modulates hormone status to favor an environment that can antagonize estrogen bioavailability and metabolism [48].

The unique composition of the test product relieves menstrual pain by selectively controlling the hormones that sense pain and controlling the contraction of the uterus to effectively shed the endometrium (lining) in a controlled manner, which contributes to preventing endometriosis that may develop at a later stage.

### Limitations and Future Direction of Study

Limitations of the present pilot study include the relatively small sample size without a standard controlled group. While the study demonstrated statistically significant menstrual pain relief between the intervention and placebo groups, to strengthen the evidence base, future studies should address these limitations by incorporating a larger sample size, a longer duration, and include a standard control group in the study design, which would provide more robust evidence, improve the generalizability of the findings, and confirm the efficacy of the intervention in a broader population.

## 5. Conclusions

Total pain relief and pain intensity difference evaluated by a pain relief score and a numerical rating scale showed that turmeric–boswellia–sesame formulation was significantly better than placebo. Mean TOTPAR and SPID at 6 h of treatment group, respectively, were 12.6 and 20.19 times better than the placebo group. The findings of the study recommend turmeric–boswellia–sesame formulation as a natural and safe alternative for menstrual pain relief.

## Figures and Tables

**Table 1:** Demographics and other baseline characteristics.

Parameters	Placebo			Treatment		
	Mean	SD	SE	Mean	SD	SE
Age (years)	26.40	4.55	0.83	26.67	4.26	0.78
Height (cm)	156.31	5.17	0.94	155.30	4.34	0.79
Weight (kg)	54.07	4.95	0.90	54.10	5.27	0.96
Duration of menstrual cycle (days)	28.50	2.71	0.50	29.27	2.49	0.45

**Table 2:** Total pain relief using PRS.

Variable	Count	Mean	SD	SE	95% LCL (Mean)	95% UCL (Mean)	*p*-Value
Placebo	30	1.5	2.11	0.39	0.71	2.29	*p* < 0.001 *
Treatment	30	18.9	3.08	0.56	17.75	20.05	

* Mann–Whitney U or Wilcoxon rank sum test for difference in location.

**Table 3:** Responder profile for % max TOTPAR.

Category (% Max TOTPAR)	Placebo	Treatment	Placebo (%)	Treatment (%)
<30	20	0	66.67	0
30–49	4	0	13.33	0
50–69	1	6	3.33	20
≥70	5	24	16.67	80

**Table 4:** Sum of pain intensity difference (SPID) at 6 h using numerical rating scale.

SPID (0–6 h)	Treatment (*n* = 30)	Placebo (*n* = 30)
Mean	34.317	1.7
SD	7.7454	3.0727
95% CI LCL (Mean)	31.4245	0.5526
95% CI UCL (Mean)	37.2088	2.8474
Mean Difference (Placebo treatment)	−32.6	
SE (Mean Diff)	1.52132	
95% CI LCL (Mean Difference)	−35.66192	
95% CI UCL (Mean Difference)	−29.57141	
p 2-sided *	<0.0001	

* Mann–Whitney test.

**Table 5:** Least-square-mean pain intensity difference from baseline of treatment and placebo using NRS.

Time	Placebo					Treatment					LSM Difference (P-T)	* p-Value
	LSM	SE (LSM)	95% CI		*p*-Value	LSM	SE (LSM)	95% CI		*p*-Value		
			LL	UL				LL	UL			
0.5	0.2333	0.2079	−0.1789	0.6455	1.0000	2.7	0.2079	2.2878	3.1122	<0.0001	−2.4667	<0.0001
1	0.3333	0.2079	−0.0789	0.7455	1.0000	3.7667	0.2079	3.3545	4.1789	<0.0001	−3.4333	<0.0001
1.5	0.2333	0.2079	−0.1789	0.6455	1.0000	4.6	0.2079	4.1878	5.0122	<0.0001	−4.3667	<0.0001
	0.2	0.2079	−0.2122	0.6122	1.0000	5.2667	0.2079	4.8545	5.6789	<0.0001	−5.0667	<0.0001
2.5	0.2667	0.2079	−0.1455	0.6789	1.0000	5.7667	0.2079	5.3545	6.1789	<0.0001	−5.5	<0.0001
3	0.2	0.2079	−0.2122	0.6122	1.0000	6.1667	0.2079	5.7545	6.5789	<0.0001	−5.9667	<0.0001
3.5	0.3333	0.2079	−0.0789	0.7455	1.0000	6.4667	0.2079	6.0545	6.8789	<0.0001	−6.1333	<0.0001
4	0.3333	0.2079	−0.0789	0.7455	1.0000	6.6333	0.2079	6.2211	7.0455	<0.0001	−6.3	<0.0001
4.5	0.3333	0.2079	−0.0789	0.7455	1.0000	6.7333	0.2079	6.3211	7.1455	<0.0001	−6.4	<0.0001
5	0.3	0.2079	−0.1122	0.7122	1.0000	6.7667	0.2079	6.3545	7.1789	<0.0001	−6.4667	<0.0001
5.5	0.3667	0.2079	−0.0455	0.7789	1.0000	6.8667	0.2079	6.4545	7.2789	<0.0001	−6.5	<0.0001
6	0.2667	0.2079	−0.1455	0.6789	1.0000	6.9	0.2079	6.4878	7.3122	<0.0001	−6.6333	<0.0001

NRS = Numerical rating scale; LSM = least square mean; * *p*-value, 2-sided Bonferroni; P = placebo; T = treatment.

**Table 6:** Responders profile for % max SPID.

Category	Placebo	Treatment	Placebo (%)	Treatment (%)
<30	30	0	100	0
30–49	0	1	0	3.33
50–69	0	6	0	20
≥70	0	23	0	76.67

**Table 7:** Global evaluation assessment.

	Poor (=0)	Fair (=1)	Good (=2)	Very Good (=3)	Excellent (=4)	Mean ± SD	Mean Difference ± SE	*p*-Value
Treatment, n (%)	0	0	0	8 (26.7%)	22 (73.3%)	3.73 ± 0.45	−3.57 ± 0.11	<0.0001
Placebo, n (%)	25 (83.3%)	5 (16.7%)	0	0	0	0.17 ± 0.38		

## Data Availability

The datasets used and/or analyzed during the current study are available from the corresponding author upon reasonable request.
